# Is There a Rise in the Importance of Socioemotional Skills in the Labor Market? Evidence From a Trend Study Among College Graduates

**DOI:** 10.3389/fpsyg.2020.01710

**Published:** 2020-07-16

**Authors:** Jim Allen, Barbara Belfi, Lex Borghans

**Affiliations:** School of Business and Economics, Maastricht University, Maastricht, Netherlands

**Keywords:** socioemotional skills, skill requirements, college graduates, labor market, trend analysis, wages

## Abstract

In this study, we examine whether socioemotional skills have become more important in the labor market within the past 14 years. To this end, we analyze data from a unique dataset on recent graduates from Dutch professional colleges (*N* = 67,000). Two different indicators of skill change are investigated, namely changes in the skill level required in the labor market and changes in the wage returns to these skills. The results indicate that socioemotional skills related to knowledge and innovation such as logical reasoning and information gathering, as well as skills related to working to plan and collaboration, have undergone a significant increase in terms of labor market requirements. We also observe an increase in the required level of the work-related skills digital literacy and occupation-specific knowledge. However, significant increases in wage returns are only observed for socioemotional skills related to knowledge and innovation. The labor market importance of socioemotional skills appears to be only modestly affected by business cycle effects.

## Introduction

The rapid development of information communication technologies (ICT) in recent decades has had far-reaching implications for how we act and interact in our daily lives, especially at work. Many routine tasks have been taken over by computers and robots, as these machines can carry out the same type of tasks at lower costs and greater levels of quality and safety. At the same time, demand has increased for non-routine tasks that cannot be specified and defined through a clear set of coded rules and are in need of human coordination and interpretation (Autor et al., 2003; Autor et al., 2006; Goos and Manning, 2007; OECD, 2018a). This line is slowly shifting, as advances in machine learning and big data use have begun to make some non-routine tasks susceptible to automation as well (Brynjolfsson et al., 2018; Ghislieri et al., 2018). Nonetheless, it is widely believed among ICT experts that certain engineering bottlenecks will remain (Robotics-VO, 2013). These bottlenecks can be divided into three types of non-routine tasks: (1) perception and manipulation tasks, i.e., tasks that require manual acuity, (2) creative intelligence tasks, i.e., tasks that require the generation of novel and useful ideas, and (3) social intelligence tasks, i.e., tasks that require social insight (Damian et al., 2017).

The alterations in job content have led to a decrease in the demand for low and especially middle skilled jobs, as many of these jobs consist to a high degree of routine tasks (Autor, 2019). The consequences for the demand for higher skilled workers seem to be more favorable, but also for this group the technical improvements have had serious implications for their work life, as they now must continuously adjust their work tasks to the rapid technological advances. As such, it has been argued that a specific set of skills has gained in importance which enable workers to adjust to these task changes (Voogt and Roblin, 2012; OECD, 2018a; Simmering et al., 2019). This shift in the importance of skills for higher skilled workers is the focus of this paper.

Skills mentioned in the literature as potentially gaining in importance include social skills, self-regulatory skills and knowledge-acquisition skills. These skills have also been referred to as socioemotional skills (Kautz et al., 2014; De Fruyt et al., 2015; OECD, 2018b). The OECD (2018b) defines socioemotional skills as abilities that allow one to manage one’s own thoughts, emotions and behavior. These skills differ from cognitive abilities such as literacy or numeracy in that they are more related to the ability to regulate oneself than to a raw ability to process information. As with closely related concepts such as non-cognitive skills, employability skills, 21st-century skills and soft skills, socioemotional skills are seen as abilities that are at least partially independent of cognitive aptitude, and that cannot be easily substituted by technology (Borghans et al., 2008; Kyllonen et al., 2014; OECD, 2018b).

During the last decade, several studies have been carried out to investigate the changing importance of socioemotional skills in the labor market. These studies have been mainly conducted from the point of view of shifts in the occupational structure. Based on the skill requirements in a baseline year, some of these studies investigated whether the number of workers in occupations that demanded more socio-emotional skills grew faster than in occupations that required mainly manual, routine or cognitive skills (Acemoglu and Autor, 2011). Others have looked at whether wages have grown faster in occupations requiring higher levels of socioemotional skills than other occupations (Castex and Dechter, 2014; Beaudry et al., 2016; Deming, 2017). For all these studies, the Dictionary of Occupational Titles (DOT) or the Occupational Information Network (O^∗^NET) have been important sources for the classification of the skill requirements (Acemoglu and Autor, 2011; Castex and Dechter, 2014; Beaudry et al., 2016; Deming, 2017). It is very likely that the changes in the labor market also changed the skill requirements within each occupation. However, as the occupational titles of the DOT and O^∗^NET are not measured repetitively over time (e.g., the last revision of O^∗^NET was in 2010), changes within occupations cannot be observed. It can therefore be argued that these studies have underestimated shifts in the demand for socioemotional skills.

Using unique data on recent graduates from Dutch professional colleges for the period 2003–2017, the present paper investigates whether the importance of socio-emotional skills in the labor market has changed for this specific group of higher educated graduates over the past fourteen years. We do so by analyzing changes in two indicators of the importance of skills: (1) skill requirements in the labor market and (2) wage returns to skills. To our knowledge, to date only Edin et al. (2018) have studied trends in the demands for socioemotional skills of individual workers – as opposed to trends in skills demands for occupation categories. Since these authors focused on relatively experienced workers, they potentially confounded learning effects in education with experience effects in the labor market. They also used an overall socioemotional score which did not distinguish between different types of socioemotional skill. By contrast, our study analyses trends in a range of socioeconomic skills among a homogeneous group of college graduates surveyed one to two years after entering the labor market. Moreover, our study also analyses wage returns to skills, thereby painting a more precise picture in terms of the changes that have been taking place in the labor market for socioeconomic skills in different domains.

In sum, the present study aims to investigate the following research questions:

1. Has the level of socioemotional skills required of higher educated workers increased in the past decade?
2. If so, has the required level of all socioemotional skills risen to the same degree?
3. To what extent have changes in the socioemotional skills required of higher educated workers been reflected in higher wage returns?

## Materials and Methods

### Participants and Procedure

The present study uses data from the HBO-Monitor, an annual survey among graduates from Dutch universities of applied sciences (henceforth professional colleges), who were surveyed one to two years after graduation. Professional colleges are one of the two main types of higher education in the Dutch binary system, the other being academic universities. While academic universities have a stronger focus on scientific research, professional colleges are more practical and vocationally oriented. Professional college graduates account for a large and highly important part of the new entrants to the labor market each year. Numerically, they outnumber graduates from academic universities by a factor of around two to one, and they are present in large numbers in almost every firm and organization of any size in the country. As a population to study changes in skill requirements over time, particularly professional colleges are interesting as, in contrast to academic universities, they aim to provide their graduates with the occupation-specific skills needed to perform at a high level in the labor market. At the same time, these colleges make strong use of precisely the kind of group-based learning methods that one might expect to be effective in fostering socioemotional skills.

The HBO-Monitor survey has been conducted annually by the Research Centre for Education and the Labour Market (ROA) of Maastricht University starting in the early 1990s and continuing to the current time. It provides information on graduates’ first experiences in the labor market, as well as the skill requirements in their job and background information such as age, gender and migration background. Around 25,000 graduates participate in this survey each year, with a response rate of about 40%. Due to changes in the questions asked, we restricted our analyses to data from the survey years 2004 through 2017, comprising cohorts who graduated in the study years 2002–2003 through 2015–2016.

### Further Selection of Cases for Present Study

As previously mentioned, the analyses in the present study focus on changes in required skill level and the wage returns to required skills of recent graduates of bachelor programs at professional colleges in the Netherlands. To ensure that the comparison over time is as consistent as possible, it is important to focus on graduates who have followed a more or less standard path through education and the initial transition to the labor market. This is because non-standard career paths may introduce variance in both skills and outcomes that is driven by different experiences than those of young graduates who proceed directly to the labor market after graduation. For this reason, the present analyses are limited to graduates who were aged 30 or less at the time of the survey and who were enrolled in full-time study programs.

The analyses of required skill level, as well as the analyses of wage returns to required skill, are by necessity restricted to graduates who were in paid employment at the time of the survey. We specifically focus on full-time graduate jobs, which we define as jobs for at least 32 h per week requiring a college degree or higher in the discipline for which the graduates were trained. Finally, graduates who were working abroad at the time of the survey are also removed from the analyses, to avoid confounding our results with effects of differences in costs of living and exchange rates. After applying all these restrictions, we are left with around 67,000 cases for analysis, an average of almost 5,000 cases per year.

### Measures

#### Required Skill Level

This variable is measured among recent college graduates by means of the following question: ‘Below you find a number of aspects that may be important for carrying out a job. For each aspect, please indicate the level required for your current job’ Each aspect was rated on a 5-point Likert scale, ranging from ‘basic’ to ‘excellent.’ The same list of skills was presented to successive graduate cohorts each year from 2004 through 2017. In total, nine socioemotional skills and for comparison three more general work-related skills were investigated. For expositional purposes, the selected socioemotional skills can be grouped into three broad categories. The first category refers to skills whose primary function is to help people regulate their own functioning at work on a day to day basis, which we henceforth refer to as self-regulation skills: (a) the ability to perform your work without supervision (WORKING INDEPENDENTLY), (b) the ability to work within a budget, plan or guideline (WORKING TO PLAN), and (c) the ability to recognize problems and opportunities (ALERTNESS). The second category of skills are deployed to help people function at work in relation to their co-workers, which we refer to as social skills: (a) the ability to cooperate productively with others (COLLABORATION) and (b) the ability to make your meaning clear to others (EXPLAINING). The third category is referred to as knowledge and innovation skills: (a) the ability to gather information (INFORMATION GATHERING), (b) the ability to come up with new ideas and solutions (CREATIVITY), (c) the ability to learn new things (LEARNING ABILITY), and (d) the ability to reason logically (LOGICAL REASONING). In addition to these nine socioemotional skills, the list contains three more general work-related skills, namely (a) knowledge of one’s own field or discipline (OCCUPATION-SPECIFIC KNOWLEDGE), (b) knowledge of other fields or disciplines (INTERDISCIPLINARY KNOWLEDGE), and (c) the ability to use information and communication technology (DIGITAL LITERACY).

#### Hourly Earnings

Respondents were asked about their monthly earnings and the working hours in their main job according to the working contract, which are used to construct a measure of log hourly earnings.

#### Control Variables

Additionally, respondents were asked about general background characteristics such as their gender, age, ethnic background (i.e., country of birth of themselves and their parents), field of study, and region of employment. In all analyses, these background characteristics are used as control variables. These control variables are centered around the grand mean, to avoid large impacts on the main effects of skills.

#### Correction for the Business Cycle

**FIGURE 1:**
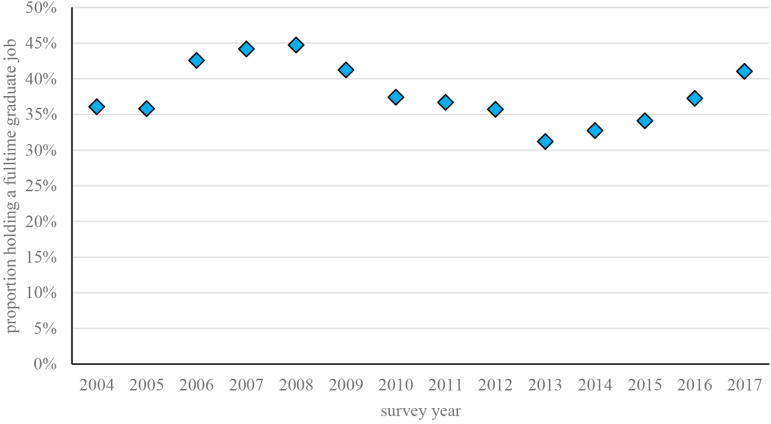
Business cycle fluctuations in graduate jobs, 2004–2017 (total *N* = 147,540).

It is possible that employers are forced to lower their standards with respect to the skills they expect graduates to have in economic good times, because they would otherwise face problems in hiring enough workers. For that reason, we include the annual percentage of graduates working full-time in graduate jobs (jobs for at least 32 hours per week requiring a college degree or higher in the discipline for which they were trained) as a business cycle indicator to the analyses. Since there are almost no regional differences in the business cycle, we apply it at the national level. Figure 1 shows the changes in this indicator over the period in question. As the figure shows, this proportion of graduates working full-time in graduate jobs rose sharply in the years before the 2008 crisis, then dropped equally sharply starting in 2009 as the crisis took its toll. From 2014 onward the proportion once again started to rise, as the graduate labor market recovered from the effects of the crisis.

## Analytical Approach

In order to determine whether the importance of socioemotional skills is rising for recent Dutch college graduates, we perform two distinct series of analyses.

First, to examine whether the required level of these skills has changed in the past 14 years, ordered probit regression analyses are employed, in which the mean level and trend in required level of each skill is estimated for graduates working in the Netherlands in college-level, full-time jobs matching the discipline for which they were trained. To ensure that substantive changes in required skills by the labor market are not confounded by shifts in the composition of the graduate population, we control for gender, age, migration background, a broad classification for field of study and region of current work. As such, these analyses are performed in two steps. First, we estimate the probit regression with dummies for each year, allowing us to plot the non-parametric development of skills requirements. Next, we estimate the model with a linear time trend to get an estimate of the average change in skills requirements of time. Since skills requirements could be sensitive to the business cycle, we also add the business cycle indicator as a control variable.

In these analyses, we implement a small departure from what is typically used in ordered probit models, where the distribution of the residuals is standardized with a mean of 0 and standard deviation of 1. Normally, the mean skill requirement in 2004 would be set equal to 0. However, since in our data the level of the average response is relevant information to take into account, we set the midpoint of the range of possible responses (the middle of the interval indicated by response option 3) equal to 0. Consequently, the constant term in the ordered probit shows how many standard deviations the mean of the distribution exceeds this midpoint. Our first probit regression can therefore be specified as follows:

$$y_{i,t}=\sum_{t=2004}^{2017}\delta_{t}\,d_{t}+X\,\beta +\varepsilon_{i,t}\,\mathrm{with}\,V\,a\,r\,\left(\varepsilon_{i,t}\right)=1$$

with

$$Y_{i,t}=1\,i\,f\,y_{i,t}<\theta_{1}$$

$$Y_{i,t}=2\,i\,f\,y_{i,t}\geq \theta_{1}\,a\,n\,d\,y_{i,t}<-\theta_{2}$$

$$Y_{i,t}=3\,i\,f\,y_{i,t}\geq -\theta_{2}\,a\,n\,d\,y_{i,t}<\theta_{2}$$

$$Y_{i,t}=4\,i\,f\,y_{i,t}\geq \theta_{2}\,a\,n\,d\,y_{i,t}<\theta_{3}$$

$$Y_{i,t}=5\,i\,f\,y_{i,t}\geq \theta_{3}$$

in which *y*_*i,t*_ is a latent variable representing the skill requirement for individual *i* observed in year *t*. *Y*_*i*,*t*_is the observed answer on the skills requirement question, ranging from 1 to 5. *d*_*t*_ indicates year dummies, equal to 1 if year = *t*, and otherwise 0. *X* represents the control variables. The θs represent the cut-off parameters. The difference with respect to the regular specification of probit is that there are no independent cut-offs points to separate answer 2 from 3 and 3 from 4. Instead we force the second cut-off to be minus the third cut-off. Now, as already indicated, the estimate of the 2004-dummy reveals the number of standard deviations of the error term by which the average skill requirement in 2004 deviates from the midpoint of the scale. Since in our data there are idiosyncratic shocks per year, we estimated the probit regression with clustered standard errors per year.^1^

In order to estimate linear trends in skill requirements we specified the following probit model:

$$y_{i,t}=c+\delta \left(t-2004\right)+\alpha B_{t}+X{\beta +\varepsilon }_{i,t}\text{with}\;Var\left(\varepsilon_{i,t}\right)=1$$

with

$$Y_{i,t}=1\,i\,f\,y_{i,t}<\theta_{1}$$

$$Y_{i,t}=2\,i\,f\,y_{i,t}\geq \theta_{1}\,a\,n\,d\,y_{i,t}<-\theta_{2}$$

$$Y_{i,t}=3\,i\,f\,y_{i,t}\geq -\theta_{2}\,a\,n\,d\,y_{i,t}<\theta_{2}$$

$$Y_{i,t}=4\,i\,f\,y_{i,t}\geq \theta_{2}\,a\,n\,d\,y_{i,t}<\theta_{3}$$

$$Y_{i,t}=5\,i\,f\,y_{i,t}\geq \theta_{3}$$

This model replaces the year dummies with a linear trend, and includes the business cycle indicator *B*_*t*_ to control for possible effects of the business cycle on skill requirements. Finally, to examine the extent to which changes in skill requirements are reflected in their returns in the labor market, we estimate additional models with log hourly earnings as dependent variable.

The wage regression is specified as follows:

$$\text{ln}\,{\left(w\right)}_{i,t}=\gamma_{1}\,Y_{i,t}+\gamma_{2}\,Y_{i,t}\,\left(t-2004\right)+\gamma_{3}\,Y_{i,t}\,B_{t}+\sum_{t=2004}^{2017}\delta_{t}\,d_{t}+X\,\beta +\varepsilon_{i,t}$$

γ_*1*_represents the wage premium of skill *y* in 2004. γ_*2*_ represents the trend in this premium from 2004 onward and γ_*3*_ how much the relationship between wage and skill requirements in affected by the business cycle. A full set of yearly dummies corrects for annual fluctuations in the average wage level. Again, the standard errors are calculated based on a clustering per year.

## Results

### Descriptive Analyses

Table 1 provides a descriptive overview of the measures used for the group of graduates included in the analyses. A little over half of included graduates are females, and the average age is 24.5 years. Most graduates are native Dutch, but a total of around 11% of graduates were either born abroad or born in the Netherlands to foreign-born parents. Almost half of the graduates worked, at the time of the survey, in the heavily populated western part of the country. Average hourly earnings over the whole period were around 14.82 euros at 2017 rates.

**TABLE 1:** Description of control and outcome variables used in the analyses.

	%	Mean	S.D.	N of observations
Gender: % female	51.6%	–	–	68,268
Age (average years)	–	24.5	1.9	68,398
Migration background (%):				66,914
*Native Dutch*	89.2%	–	–	
*Non-western migration background*	4.9%	–	–	
*Western migration* *background*	5.9%	–	–	
Region of work (%)				66,920
*North*	8.1%	–	–	
*East*	20.6%	–	–	
*West*	48.6%	–	–	
*South*	22.7%	–	–	
Hourly earnings (average 2017 euros)	–	14.83	3.02	61,609

Table 2 shows the mutual correlations between required skills, age and hourly earnings for graduates working in the Netherlands in fulltime jobs matching their level and field of education. In general, most of the skills are quite strongly related to each other in terms of required level. The strongest exception is for INTERDISCIPLINARY KNOWLEDGE, which is only moderately related to the other skills in terms of required level. The other work-related skills – OCCUPATION-SPECIFIC KNOWLEDGE and DIGITAL LITERACY – are also a little less strongly related to the socioemotional skills than the socioemotional skills are related to each other. There are particularly strong correlations between CREATIVITY and LEARNING ABILITY (*r* = 0.49, *p* < 0.01), between ALERTNESS and INFORMATION GATHERING (*r* = 0.47, *p* < 0.01), between EXPLAINING and LEARNING ABILITY (*r* = 0.46, *p* < 0.01), and between ALERTNESS and LOGICAL REASONING (*r* = 0.45, *p* < 0.01). Most of the required skills show weak but significant negative correlations with age, and weak but significant positive relations with real hourly earnings. Age is strongly correlated with real hourly earnings (*r* = 0.18, *p* < 0.01).

**TABLE 2:** Correlations between required skills, age and real hourly earnings.

	Working independ-ently	Working to plan	Alertness	Explaining	Collabor-ation	Informat-ion gathering	Creativity	Learning ability	Logical reasoning	Occupation-specific knowledge	Interdisc-iplinary knowledge	Digital literacy	Age
Working to plan	0.233												
Alertness	0.364	0.288											
Explaining	0.445	0.263	0.461										
Collaboration	0.341	0.249	0.353	0.427									
Information gathering	0.304	0.275	0.474	0.364	0.285								
Creativity	0.342	0.267	0.436	0.441	0.360	0.336							
Learning ability	0.355	0.259	0.393	0.460	0.395	0.385	0.494						
Logical reasoning	0.382	0.337	0.452	0.427	0.340	0.424	0.393	0.408					
Occupation-specific knowledge	0.286	0.201	0.343	0.325	0.265	0.287	0.304	0.336	0.327				
Interdisciplinary knowledge	0.140	0.175	0.227	0.187	0.167	0.238	0.238	0.234	0.208	0.233			
Digital literacy	0.210	0.243	0.277	0.244	0.222	0.388	0.249	0.276	0.297	0.215	0.213		
Age	−0.040	0.005	−0.049	−0.050	−0.059	−0.006	−0.044	−0.054	−0.025	−0.062	0.006	0.001	
Real hourly earnings	0.018	0.016	0.035	0.040	-0.007	0.044	0.001	0.019	0.036	0.042	0.011	0.016	0.175

*Number of cases varies per correlation, between 59,968 and 68,398.*

Table 3 shows how required skills, age and hourly earnings are related to gender, migration background, broad field of study and region of work. The between-group differences are generally quite small, but being based on a large number of cases nonetheless mostly statistically significant. In general, women report a slightly higher level of required skill than men, with DIGITAL LITERACY the notable exception to the rule, with men reporting a higher required level of that skill than women. There is little difference in required skill by migration background, with DIGITAL LITERACY once again the main exception. Graduates with (non-Dutch) western and non-western migration backgrounds report a somewhat higher required level of DIGITAL LITERACY than native Dutch graduates. There is relatively little systematic difference between fields of study in required skill levels, although there are some specific skills that score relatively high or low in certain fields. For example, teaching and education graduates work in jobs typically requiring a high level of OCCUPATION-SPECIFIC KNOWLEDGE but a relatively low level of WORKING TO PLAN. There is very little variation in required skill level by region of work.

**TABLE 3:** Mean of required skills, age and real hourly earnings, by gender, migration background, broad field of study and region of work.

Geslacht	Working independ-ently	Working to plan	Alertness	Explaining	Collabor-ation	Informat-ion gathering	Creativity	Learning ability	Logical reasoning	Occupation-specific knowledge	Interdisc-iplinary knowledge	Digital literacy	Age	Real hourly earnings
**Gender**														
*Male*	4.16	3.72	4.00	4.05	3.92	3.87	3.81	3.88	4.02	3.77	3.27	3.75	24.96	15.18
*Female*	4.35	3.69	4.15	4.22	4.14	3.91	3.97	4.02	4.05	3.92	3.31	3.67	24.14	14.50
Migration background														
*Western* *(excl. native* *Dutch)*	4.27	3.76	4.10	4.17	4.08	3.94	3.92	3.97	4.08	3.83	3.32	3.81	25.07	14.95
*Non-western*	4.24	3.78	4.08	4.14	4.08	3.94	3.86	3.99	4.05	3.80	3.36	3.81	25.57	15.29
*Native Dutch*	4.26	3.70	4.08	4.13	4.03	3.88	3.89	3.95	4.03	3.85	3.29	3.70	24.43	14.79
**Broad Field of study**														
*Agriculture* * and food*	4.30	3.79	4.08	4.13	3.96	3.89	3.88	3.96	4.08	3.89	3.27	3.73	24.60	14.65
*Teaching and* *education*	4.30	3.40	4.22	4.28	4.16	3.74	4.05	4.03	3.87	4.03	3.34	3.51	24.11	14.16
*Technical*	4.16	3.74	3.95	4.00	3.94	3.86	3.86	3.92	4.06	3.75	3.23	3.77	24.77	14.98
*Economics*	4.24	3.82	4.04	4.10	3.98	3.95	3.83	3.90	4.04	3.75	3.31	3.82	24.63	14.62
*Health* *studies*	4.39	3.71	4.06	4.21	4.13	3.89	3.86	4.05	4.15	4.05	3.26	3.61	24.19	15.85
*Social* *studies*	4.37	3.67	4.31	4.29	4.18	3.97	3.98	4.00	4.07	3.91	3.36	3.56	24.68	15.28
**Region of current work**														
*North*	4.29	3.69	4.08	4.16	4.03	3.89	3.90	3.96	4.02	3.86	3.34	3.69	24.77	14.49
*East*	4.26	3.67	4.08	4.13	4.01	3.87	3.90	3.94	4.02	3.86	3.30	3.68	24.46	14.62
*West*	4.26	3.72	4.08	4.13	4.05	3.89	3.89	3.95	4.03	3.85	3.29	3.73	24.54	15.01
*South*	4.26	3.70	4.08	4.13	4.03	3.90	3.88	3.95	4.06	3.83	3.29	3.71	24.49	14.73

*a. Between-group variance is significant at *p* < 0.01 in all cases, except for working independently × migration background, alertness × migration background, working independently × region, alertness × region, explaining × region, creativity × region, and learning ability × region (*p* > 0.05). b. Number of cases varies per table, between 59,311 and 66,920.*

### Multivariate Analyses

As already outlined in the introduction, we use two indicators to assess the changing importance of socioemotional skills: (1) Changes in skill requirements and (2) changes in wage returns to skills. In this section we present the results of a series of multivariate analyses designed to provide a robust picture of these changes.

#### Which Skills Were Most Important in 2004?

Before we analyze the trends in importance of skills, it is important to identify the skills that were already relatively important at the start of the period under consideration – namely in 2004 –, and the skills that were relatively less important at that time. For a proper interpretation of trends, it makes a good deal of difference whether the changes are to skills that were already important, or to skills that were formerly less important but are becoming more so in time. Table 4 shows this. We include both measures of importance in the table. The coefficients in the table with respect to skill requirements can be interpreted as the number of standard deviations that the average respondent lies above the midpoint of the 5-point response scale in 2004. For example, in Table 4, the skill requirements coefficient of 0.690 for the skill WORKING TO PLAN, indicates that the average respondent has a required level of this skill equal to 0.690 standard deviations above the scale midpoint. The coefficients with respect to the wage effects can be interpreted as the extra wage a graduates received in 2004 who scores one point higher on the relevant skill requirement scale. For example, in Table 4, the wage coefficient 0.006 for the skill WORKING INDEPENDENTLY indicates that an increase of one scale point on this skill is associated with an increase of 0.6% in hourly wages.

**TABLE 4:** Importance of skills in 2004 (intercept).

	Required skills			Wages		
		
	*b*	*P*	*N*	*b*	*p*	*N*
	(*SE*)			(*SE*)		
**Self-regulation skills**						
*Working independently*	1.694	**	63,833	0.006	**	57,778
	(0.032)			(0.001)		
*Working to plan*	0.690	**	64,429	0.003		58,298
	(0.016)			(0.002)		
*Alertness*	1.336	**	64,980	0.015	**	58,757
	(0.035)			(0.003)		
**Social skills**						
*Explaining*	1.504	**	64,036	0.017	**	57,963
	(0.029)			(0.002)		
*Collaboration*	1.269	**	63,885	0.002		57,827
	(0.011)			(0.002)		
**Knowledge and innovation skills**						
*Information gathering*	1.145	**	64,979	0.006	**	58,752
	(0.015)			(0.001)		
*Creativity*	1.033	**	64,113	0.004	*	58,024
	(0.023)			(0.002)		
*Learning ability*	1.161	**	64,082	0.004	**	58,000
	(0.024)			(0.001)		
*Logical reasoning*	1.360	**	64,448	0.007	**	58,291
	(0.022)			(0.002)		
**Work-related knowledge and skills**						
*Occupation-specific knowledge*	1.088	**	65,588	0.014	**	59,250
	(0.023)			(0.001)		
*Interdisciplinary knowledge*	0.383	**	65,215	0.006	**	58,940
	(0.038)			(0.002)		
*Digital literacy*	0.796	**	64,981	0.002	*	58,755
	(0.021)			(0.001)		

* *p* < 0.05, ** *p* < 0.01. *Note.* All analyses include controls for gender, age, broad field of study and region of work f (see Supplementary Appendix A, Tables A1, A2 for details). The stars that indicate the level of significance have to be put closer to the parameters.

All of the coefficients for required skills are significantly positive, indicating that the majority of respondents have a required level of skill of greater than 3, which is the midpoint of the scale. Most of the coefficients for the effect of skills on wages are significantly positive, the exceptions being WORKING TO PLAN and COLLABORATION. Looking at the size and rank order of the coefficients, there is some overlap in the ranking of importance in 2004 according to the two indicators, but also some differences. For example, EXPLAINING and ALERTNESS were not only characterized by a relatively high required level, but also yielded relatively high wage returns. At the other end of the continuum, WORKING TO PLAN, INTERDISCIPLINARY KNOWLEDGE, CREATIVITY and DIGITAL LITERACY emerge as relatively less important in 2004 in terms of both skill requirements and wage returns. On the other hand, WORKING INDEPENDENTLY and COLLABORATION show low or even non-significant wage returns despite being required at a relatively high level, while OCCUPATION-SPECIFIC KNOWLEDGE yields high wage returns despite only being required at a relatively modest level. These differences illustrate the fact that skills that are required at a high level in graduate jobs are not always the skills that employers are prepared to pay the most for.

#### Which Skills Are Becoming More Important Over Time?

**FIGURE 2:**
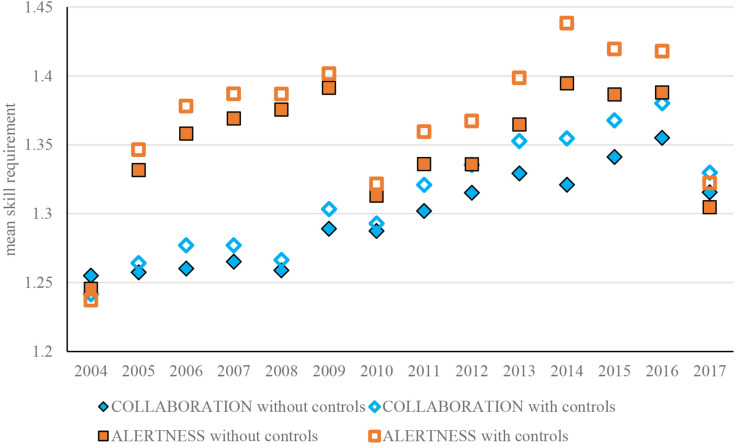
Estimated required level of the skills collaboration and alertness per year, before and after adding controls*. Notes: 1.*Controls included for gender, age, broad field of study, region of work and GPA. 2.*Number of cases collaborations = 63,189; number of cases alertness = 64,564.

In order to gain a first impression of changes over time in the importance of skills, we can look at changes from year to year in mean requirements and wage returns to skills. To ensure that trends are not confounded by changes from year to year in the composition of the graduate population, we control for gender, age, migration background, a broad classification for field of study and region of current work. To illustrate how this impacts the estimates, Figure 2 shows the changes over time in the required level of the skills COLLABORATION and ALERTNESS, before and after adding the control variables.

In general, the trends appear to become somewhat stronger after controlling for the composition of the graduate population. This appears to be mainly due to the fact that the mean age has increased in the same period by around three quarter of a year, with age being negatively correlated with the required level of most of the skills (see Table 2). There have also been significant changes in composition in terms of migration background, broad field of study and region of work, confirming the importance of controlling for these variables.^2^

**FIGURE 3:**
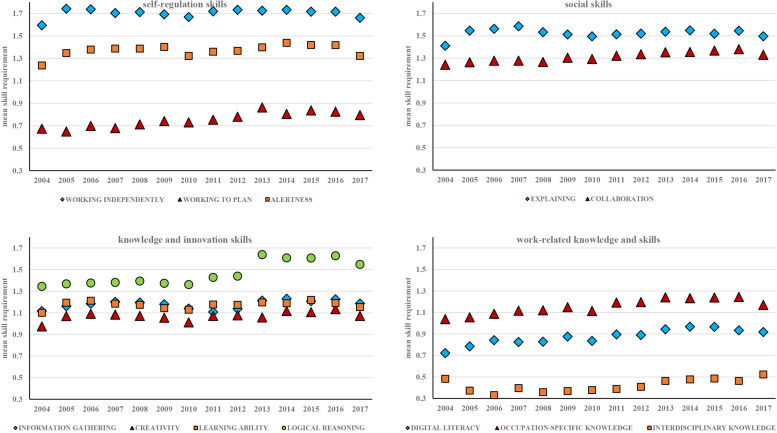
Changes in required level of skills, 2004–2017. Notes: 1. All analyses include controls for gender, age, broad field of study, region of work and GPA. 2. Number of cases varies per dimension of required skill, between 63,456 and 65,113.

Although the controls lead to somewhat stronger trends, the general pattern is similar before and after adding the controls. In the interests of brevity, we therefore subsequently only show the estimated trends after adding controls. Figure 3 shows the changes over time in the estimated required level of all 12 skills, grouped into the four skill clusters.

For some skills, such as LOGICAL REASONING, OCCUPATION SPECIFIC KNOWLEDGE, DIGITAL LITERACY, WORKING TO PLAN and COLLABORATION, there seems to be a discernible increase in the level of requirements over time. For other skills, such as INTERDISCIPLINARY KNOWLEDGE, ALERTNESS, CREATIVITY and INFORMATION GATHERING, there seems to be some increase over time, but it is harder to distinguish this from the non-linear fluctuations from year to year. For the remaining skills there is no discernible trend at all.

A problem with this purely descriptive approach is that is does not provide any guidance on how to interpret changes, even when these changes are clear and systematic. Changes over time could reflect not only a trend toward more importance, but could also be affected by, for example, changing conditions in the graduate labor market. In particular, business cycle fluctuations may not only affect graduates’ chances of finding employment, but also the kinds of skills they are required to possess, and the degree to which those skills are rewarded in the form of higher wages. This is an importance issue in this instance, since one of the most serious economic recessions in history took place in the middle of the period under consideration.

In order to obtain a clear and robust picture of the extent to which the importance of skills has changed systematically over time, the business cycle indicator was added to the model.

In the new model specification, the coefficient of the trend indicator allows us to precisely quantify the size and significance of systematic changes in skill requirements over time. By interacting the trend with skills in the wage models, we can also precisely quantify any systematic change in wage effects of skills over time. By controlling for business cycle fluctuations, we are able to obtain an estimate of that part of these trends left when the effects of the economic cycle have been taken into account. Table 5 shows these trends.

**TABLE 5:** Trends in the importance of skills.

	Required skills			Wages		
		
	*b*	*P*	*N*	*b*	*p*	*N*
	(*SE*)			(*SE*)		
**Self-regulation skills**						
*Working independently*	0.001		63,833	0.0002		57,778
	(0.004)			(0.0002)		
*Working to plan*	0.013	**	64,429	0.0002		58,298
	(0.002)			(0.0002)		
*Alertness*	0.005		64,980	−0.0002		58,757
	(0.005)			(0.0004)		
**Social skills**						
*Explaining*	0.002		64,036	−0.0003		57,963
	(0.004)			(0.0003)		
*Collaboration*	0.008	**	63,885	0.0002		57,827
	(0.002)			(0.0003)		
**Knowledge and innovation skills**						
*Information gathering*	0.005	*	64,979	0.0007	**	58,752
	(0.002)			(0.0002)		
*Creativity*	0.006		64,113	0.0003		58,024
	(0.003)			(0.0002)		
*Learning ability*	0.002		64,082	0.0007	**	58,000
	(0.003)			(0.0001)		
*Logical reasoning*	0.021	**	64,448	0.0007	**	58,291
	(0.002)			(0.0002)		
**Work-related knowledge and skills**						
*Occupation-specific knowledge*	0.013	**	65,588	0.0002		59,250
	(0.003)			(0.0001)		
*Interdisciplinary knowledge*	0.008		65,215	−0.0002		58,940
	(0.005)			(0.0002)		
*Digital literacy*	0.014	**	64,981	0.0005	**	58,755
	(0.003)			(0.0002)		

*p < 0.05, **p < 0.01. *Note.* All analyses include controls for gender, age, broad field of study and region of work f (see Supplementary Appendix A, Tables A1, A2 for details). The stars that indicate the level of significance have to be put closer to the parameters.

As was the case with the importance of skills at the start of the period under consideration in 2004, there is some overlap between skill requirements and wage effects in terms of the trend in importance, but also some clear differences. According to both measures we see strong positive trends in the importance of LOGICAL REASONING and DIGITAL LITERACY. INFORMATION GATHERING is also gaining importance according to both measures, more strongly in the case of wage effects than in terms of skill requirements. Interestingly, despite showing no significant increase in importance in terms of skill requirements, the wage effects of LEARNING ABILITY are increasing strongly over time. Conversely, we see no increase in wage returns to OCCUPATION-SPECIFIC KNOWLEDGE, WORKING TO PLAN, COLLABORATION, CREATIVITY and INTERDISCIPLINARY KNOWLEDGE, despite significant increases in requirements over time for these skills.

#### Which Skills Vary in Importance According to Economic Circumstances?

Finally, the trends have been corrected for cyclical fluctuations in the graduate labor market. This is important, because it ensures that the trends we observe are real trends, and not just the result of, for example, adjustments to the economic crisis in the years after 2008. The effects of economic circumstances are also interesting in their own right. Table 6 shows the effects of the economic cycle on the trends.

**TABLE 6:** Effects of economic cycle on the importance of skills.

	Required skills			Wages		
		
	*b*	*p*	*N*	*b*	*p*	*N*
	(*SE*)			(*SE*)		
**Self-regulation skills**						
*Working independently*	−0.049		63,833	0.033		57,778
	(0.297)			(0.018)		
*Working to plan*	−0.376		64,429	0.000		58,298
	(0.201)			(0.03)		
*Alertness*	0.111		64,980	−0.023		58,757
	(0.372)			(0.035)		
**Social skills**						
*Explaining*	0.257		64,036	−0.048	*	57,963
	(0.301)			(0.021)		
*Collaboration*	−0.255	*	63,885	−0.034		57,827
	(0.102)			(0.021)		
**Knowledge and innovation skills**						
*Information gathering*	0.199		64,979	0.067	**	58,752
	(0.207)			(0.023)		
*Creativity*	0.179		64,113	−0.015		58,024
	(0.214)			(0.029)		
*Learning ability*	−0.030		64,082	0.008		58,000
	(0.21)			(0.015)		
*Logical reasoning*	−0.743	*	64,448	0.019		58,291
	(0.289)			(0.029)		
**Work-related knowledge and skills**						
*Occupation-specific knowledge*	−0.320		65,588	−0.026		59,250
	(0.251)			(0.018)		
*Interdisciplinary knowledge*	−0.435		65,215	0.002		58,940
	(0.319)			(0.023)		
*Digital literacy*	−0.159		64,981	0.046		58,755
	(0.227)			(0.034)		

*p < 0.05, **p < 0.01. *Note.* All analyses include controls for gender, age, broad field of study and region of work f (see Supplementary Appendix A, Tables A1, A2 for details). The stars that indicate the level of significance have to be put closer to the parameters.

In general, the effects of the economic cycle are not very strong, and for fully half of the skills we see no significant change as conditions in the graduate labor market change over time. The effects such as they are, are each only observed for one of the two indicators. The required level of WORKING TO PLAN, LOGICAL REASONING, and COLLABORATION become weaker as the graduate economy improves and stronger when things take a turn for the worse. The most probable explanation for this is that the jobs that are left for graduates during a recession are on average different than the jobs that are available when the economy is booming. Since we only look at graduates in jobs matching their level of education, this suggests that jobs requiring these three skills are relatively robust and these skills are relatively needed even more when demand is low.

There are no wage effects accompanying these cyclical effects in skill requirements. However, we do find positive earnings effect for INFORMATION GATHERING (*b* = 0.067, *p* < 0.01) and a negative effect for EXPLAINING (*b* = −0.048, *p* < 0.05). The positive effect indicates that INFORMATION GATHERING is more rewarded than usually during good economic years, and relatively less during a recession. The negative effect indicates that EXPLAINING is rewarded relatively more during a recession than in boom years.

## Discussion

It has been argued that rapid technological changes are increasing the importance of socioemotional skills in the labor market for highly educated graduates. In this study, we have looked for evidence of such changes in the labor market for Dutch professional college graduates during the past 14 years. This was done using a unique dataset on recent graduates from Dutch professional colleges, containing information on nine socioemotional skills, three more general job-related skills and a range of other personal and work characteristics. Two different indicators of change in the importance of skills were investigated, namely changes in the level of skill required in the graduate labor market and changes in the wage returns to these skills. In estimating these changes, we corrected for business cycle fluctuations and reported on which socioemotional skills have become more or less important in good economic times as opposed to downturns. To provide some structure to our results, the twelve skills were grouped into four categories: self-regulation skills, social skills, knowledge and innovation skills, and work-related skills.

The findings revealed that socioemotional skills related to knowledge and innovation such as logical reasoning and information gathering as well as working to plan and collaboration have increased significantly in terms employer requirements between 2004 and 2017. We also observed an increase in the required level of the work-related skills digital literacy and occupation-specific knowledge. The trends in required skill level are thus divided across all four skill categories, suggesting that college graduates are facing increasingly high demands in terms of how they regulate their own work behavior, how they interact with others in the work place, how they collect and process information, and how well they have mastered the work-related content that they need to perform their work on a day to day basis. It is worth noting that all the socioemotional skills that did not show a positive trend in terms of required level were already required at a high level in 2004. The reverse is also mostly true: most of the skills that have experienced an increase in importance were at most moderately important in 2004. As such, there is to some extent a narrowing of the gap in required level between skills, which implies that most of the socioemotional skills we examined have either already long been important in graduate employment or are becoming more important over time. In the case of logical reasoning, both are true: this skill has experienced a sharp increase in required level during the investigated period of fourteen years, even though it was already one of the skills required at the highest level in 2004.

Regarding wage returns to required skills, significant results were only found for socioemotional skills related to knowledge and innovation, as well as for the work-related skill digital literacy. Once again it is worth remarking that a number of skills that showed no positive trend in wage returns were already relatively well-rewarded in 2004. This applies to the socioemotional skills explaining and alertness, as well as to the work-related skill occupation-specific knowledge. However, several socio-emotional skills that showed relatively low wage returns in 2004 – working to plan, collaboration and creativity – showed no significant increase in wage returns over time. Interestingly, as we saw above, the first two of these were among the skills with the strongest increase in terms of required level. This suggest that these skills are indeed becoming an increasingly important feature of graduate working life, but that these changes are so far not reflected in greater wage returns.

What does this mean in concrete terms for policymakers and educational practitioners who aim to prepare college students as well as possible for today’s labor market? First of all, it means that it is very important that higher education institutions monitor the degree to which their graduates are equipped for the changing demands of the labor market and, where necessary, focus on how they can contribute to the development of socioemotional skills, especially those that are in high and/or increasing demand. Fortunately, the HBO-Monitor, the survey on which our analyses are based, is specifically designed to help professional colleges to do precisely that. Each year the colleges receive a detailed report on their own graduates, including information on the skills required in the labor market. If it appears that graduates of some study programs are failing to adapt sufficiently to changes in demands for certain socioemotional skills, there are a range of ways in which colleges can remedy this. For example, active learning methods, such as collaborative, personalized and problem-based learning, have proven effective in enhancing socioemotional skills (Laursen et al., 2013; Baepler et al., 2014). The idea behind these learning methods is that by making learning an activity that is controlled by the learners themselves – rather than a teacher or a subject expert – learners learn how to gain control of their own learning process, by determining their own learning needs, learning strategies, and learning materials, alone or together with other learners. Students can also enhance their own self-regulation and knowledge and innovation skills by formulating their own learning goals, identifying human and material learning resources, and evaluating their own learning outcomes (Tekkol and Demirel, 2018).

As mentioned before, our dataset differed from those used by many other researchers investigating changes in the importance of socioemotional skills in the labor market. This has advantages and disadvantages that are worthy of note. On the positive side, our data provided a more precise and differentiated measure of the skills that employees possess, as well as those that they need to carry out their daily jobs than earlier studies on this topic. Since the skill items were strictly comparable across the full time period studied, trends in required skill level accurately assessed. Furthermore, an extensive psychological literature base confirms that people are relatively good at using questionnaires to communicate their true experiences, conditional upon them knowing how the questions asked should be answered, and on them feeling at ease in reporting accurately on them (see for an overview, Duckworth and Yeager, 2015). As such, anonymous self-report questionnaires are better suited than other measures for assessing personal attributes.

However, there also certain disadvantages related to this approach. All our central measures are based on self-reports in questionnaire surveys. This may introduce some subjectivity regarding these measures. For example, in order to answer the questions on required skill level, some frame of reference has to be used to arrive at a certain judgment. As such, a ‘reference bias’ may occur, in which some respondents may use different standards than others. Furthermore, for various reasons, respondents may not always be entirely truthful about their stances and dispositions. A well-known example is ‘acquiescence bias,’ a tendency of people to give the average answer on all questions, either to deal with it quickly or because they have difficulty in placing themselves with respect to the average. In most respects, our survey data should not be expected to be especially prone to such measurement errors, and any bias that is present should not change systematically over time. For this reason, we believe that our assessment of trends in required skills and their relation to outcomes is basically valid, even if potentially subject to a certain amount of random “noise.” Moreover, we have strived to avoid the above-mentioned forms of bias, by guaranteeing respondents that their data would be treated anonymously, and by giving respondents the opportunity to skip certain questions they would prefer not to answer.

In sum, the present study has showed that socioemotional skills have increasingly been required and rewarded in college graduates in the labor market in the past 14 years. Particularly knowledge and innovation skills and digital literacy have become increasingly rewarded by employers, suggesting that to secure a job in the labor market of tomorrow, it is worthwhile for graduates to invest in this type of skills.

## Data Availability Statement

The datasets generated for this study are available on request to the corresponding author.

## Ethics Statement

Ethical review and approval were not required for the study on human participants in accordance with the local legislation and institutional requirements. Written informed consent from the participants was not required to participate in this study in accordance with the national legislation and the institutional requirements.

## Author Contributions

LB developed the study concept. JA, LB, and BB contributed to the study design. JA and BB were responsible for data preparation and data management. LB and JA analyzed the data, and all authors were involved in interpreting the results. BB drafted the manuscript, and all coauthors contributed to the final manuscript. All authors approved the final manuscript for submission.

## Conflict of Interest

The authors declare that the research was conducted in the absence of any commercial or financial relationships that could be construed as a potential conflict of interest.

## Footnotes

1 The results are almost identical to those of a multilevel probit with individuals nested within years.

2 The total graduate population has grown considerably in the same period, both in absolute terms and as a proportion of the working age population. Around 21% of the working age population had a college degree in 2017, compared to around 15% in 2004 (CBS, 2019). It is not immediately clear how this could impact the importance of graduate skills, but there is little reason to expect this to have systematically enhanced the importance of graduate skills.
