# Supplementary material for: Is There a Rise in the Importance of Socioemotional Skills in the Labor Market? Evidence From a Trend Study Among College Graduates
Source: Front Psychol. 2020 Jul 16;11:1710. doi: 10.3389/fpsyg.2020.01710 (PMC7392119; doi:10.3389/fpsyg.2020.01710)
Supplement: Supplementary file 1 [file Data_Sheet_1.pdf]

Supplementary Material

Is there a rise in the importance of socioemotional skills in the labor market? Evidence from a trend study among college graduates

Jim Allen, Barbara Belfi and Lex Borghans

Frontiers in Psychology – Personality and Social Psychology

doi: 10.3389/fpsyg.2020.01710

Appendix A

Table A1 Analysis of required skills, full models (part 1)

	working independ-ently		working to plan		alertness		explaining		collaboration		information gathering	
	b (*SE*)	*p*	*b* (*SE*)	*p*	*b* (*SE*)	*p*	*b* (*SE*)	*p*	*b* (*SE*)	*p*	*b* (*SE*)	*p*
Linear trend	0.001		0.013	**	0.005		0.002		0.008	**	0.005	*
	(0.004)		(0.002)		(0.005)		(0.004)		(0.002)		(0.002)	
Economic cycle	−0.049		−0.376		0.111		0.257		−0.255	*	0.199	
	(0.297)		(0.201)		(0.372)		(0.301)		(0.102)		(0.207)	
Gender (female)	0.254	**	0.061	**	0.092	**	0.119	**	0.212	**	0.075	**
	(0.007)		(0.011)		(0.013)		(0.014)		(0.012)		(0.013)	
Age	−0.006	**	−0.007	*	−0.020	**	−0.018	**	−0.022	**	−0.006	
	(0.002)		(0.003)		(0.003)		(0.003)		(0.003)		(0.004)	
Broad field of study (ref: economics)												
agriculture and food	0.022		0.168	**	−0.300	**	−0.201	**	−0.207	**	−0.072	*
	(0.047)		(0.022)		(0.044)		(0.053)		(0.031)		(0.033)	
teaching and	−0.091	**	−0.268	**	−0.125	**	−0.021		−0.007		−0.324	**
	(0.03)		(0.024)		(0.027)		(0.034)		(0.032)		(0.022)	
technical	−0.156	**	0.115	**	−0.467	**	−0.358	**	−0.204	**	−0.110	**
	(0.029)		(0.017)		(0.036)		(0.047)		(0.032)		(0.023)	
health studies	−0.104	**	0.180	**	−0.370	**	−0.260	**	−0.216	**	−0.017	
	(0.031)		(0.019)		(0.04)		(0.044)		(0.03)		(0.019)	
social studies	0.029		0.027		−0.390	**	−0.144	**	−0.039		−0.139	**
	(0.029)		(0.028)		(0.034)		(0.03)		(0.033)		(0.026)	
Region (ref: west)												
north	0.027		0.024		−0.012		0.012		−0.003		0.014	
	(0.022)		(0.019)		(0.02)		(0.013)		(0.019)		(0.016)	
east	0.000		0.002		−0.018		−0.010		−0.035	*	−0.016	
	(0.016)		(0.012)		(0.015)		(0.012)		(0.014)		(0.014)	
south	0.001		0.029	**	−0.010		−0.007		0.023	*	−0.002	
	(0.014)		(0.01)		(0.01)		(0.012)		(0.01)		(0.01)	
Migration background (ref: native Dutch)												
western (excl. native	0.007		0.043	*	0.057	*	0.086	**	0.097	**	0.064	**
	(0.029)		(0.018)		(0.026)		(0.03)		(0.029)		(0.021)	
non-western	−0.019		0.037	*	0.029		0.042	*	0.095	**	0.041	
	(0.021)		(0.017)		(0.021)		(0.018)		(0.022)		(0.025)	
Constant	1.694	**	0.690	**	1.336	**	1.504	**	1.269	**	1.145	**
	(0.032)		(0.016)		(0.035)		(0.029)		(0.011)		(0.015)	
cut 1	−1.362	**	−1.161	**	−1.317	**	−1.444	**	−1.284	**	−1.407	**
	(0.046)		(0.017)		(0.029)		(0.032)		(0.026)		(0.018)	
cut 3 = − cut 2	−0.564	**	−0.413	**	−0.520	**	−0.599	**	−0.510	**	−0.557	**
	(0.03)		(0.017)		(0.034)		(0.026)		(0.013)		(0.017)	
cut 4	1.972	**	1.499	**	1.861	**	1.963	**	1.880	**	1.995	**
	(0.039)		(0.017)		(0.039)		(0.037)		(0.016)		(0.023)	
Pseudo R^2	0.010		0.009		0.011		0.011		0.012		0.005	

Table A1 Analysis of required skills, full models (part 2)

	creativity		learning ability		logical reasoning		occupation-specific knowledge		interdisciplinary knowledge		digital literacy	
	b	*p*	*b*	*p*	*b*	*p*	*b*	*p*	*b*	*p*	*b*	*p*
	(SE)		*(SE)*		*(SE)*		*(SE)*		*(SE)*		*(SE)*	
Linear trend	0.006		0.002		0.021	**	0.013	**	0.008		0.014	**
	(0.003)		(0.003)		(0.002)		(0.003)		(0.005)		(0.003)	
Economic cycle	0.179		−0.030		−0.743	**	−0.320		−0.435		−0.159	
	(0.214)		(0.21)		(0.289)		(0.251)		(0.319)		(0.227)	
Gender (female)	0.174	**	0.147	**	0.080	**	0.063	**	0.006		0.013	
	(0.012)		(0.012)		(0.013)		(0.012)		(0.012)		(0.014)	
Age	−0.014	**	−0.023	**	−0.019	**	−0.024	**	0.003		−0.010	**
	(0.003)		(0.003)		(0.004)		(0.003)		(0.003)		(0.004)	
Broad field of study (ref:												
agriculture and food	−0.031		0.030		0.054	*	0.034		−0.091	*	0.208	**
	(0.047)		(0.043)		(0.026)		(0.051)		(0.038)		(0.036)	
teaching and education	0.128	**	0.061	*	−0.277	**	0.202	**	0.012		−0.051	
	(0.035)		(0.028)		(0.027)		(0.016)		(0.016)		(0.033)	
technical	−0.005		0.005		0.044		−0.175	**	−0.151	**	0.266	**
	(0.033)		(0.02)		(0.025)		(0.032)		(0.018)		(0.025)	
health studies	−0.114	**	−0.063	**	−0.021		−0.188	**	−0.054	**	0.312	**
	(0.041)		(0.017)		(0.027)		(0.025)		(0.018)		(0.029)	
social studies	−0.144	**	0.085	**	0.112	**	0.203	**	−0.116	**	0.056	
	(0.031)		(0.028)		(0.028)		(0.027)		(0.033)		(0.029)	
Region (ref: west)												
north	0.018		−0.003		−0.030		0.030		0.059	**	−0.008	
	(0.025)		(0.019)		(0.018)		(0.02)		(0.016)		(0.018)	
east	0.008		−0.032	**	−0.050	**	0.010		0.003		−0.022	*
	(0.012)		(0.011)		(0.018)		(0.015)		(0.009)		(0.01)	
south	0.005		−0.022	*	−0.037	**	0.030	**	−0.013		0.010	
	(0.012)		(0.011)		(0.011)		(0.01)		(0.011)		(0.013)	
Migration background (ref:												
western (excl. native	0.055	**	0.057	*	0.065	**	0.023		0.048		0.118	**
	(0.018)		(0.026)		(0.021)		(0.03)		(0.027)		(0.02)	
non-western	−0.007		0.106	**	0.038		−0.017		0.086	**	0.107	**
	(0.016)		(0.024)		(0.022)		(0.022)		(0.011)		(0.027)	
Constant	1.033	**	1.161	**	1.360	**	1.088	**	0.383	**	0.796	**
	(0.023)		(0.024)		(0.022)		(0.023)		(0.038)		(0.021)	
cut 1	−1.284	**	−1.288	**	−1.399	**	−1.310	**	−1.642	**	−1.384	**
	(0.023)		(0.021)		(0.054)		(0.022)		(0.044)		(0.015)	
cut 3 = − cut 2	−0.491	**	−0.508	**	−0.594	**	−0.528	**	−0.623	**	−0.517	**
	(0.023)		(0.024)		(0.027)		(0.022)		(0.041)		(0.021)	
cut 4	1.785	**	1.815	**	2.135	**	2.141	**	2.160	**	1.810	**
	(0.027)		(0.026)		(0.023)		(0.023)		(0.033)		(0.029)	
Pseudo R^2	0.006		0.005		0.010		0.014		0.002		0.009	

* p < 0.05, ** p < 0.01.

Table A2 Analysis of effects on wages, full models (part 1)

	working independently		working to plan		alertness		explaining		collaboration		information gathering	
	b (*SE*)	*p*	*b* (*SE*)	*p*	*b* (*SE*)	*p*	*b* (*SE*)	*p*	*b* (*SE*)	*p*	*b* (*SE*)	*p*
Required skill	0.006	**	0.003		0.015	**	0.017	**	0.002		0.006	**
	(0.001)		(0.002)		(0.003)		(0.002)		(0.002)		(0.001)	
Linear trend	0.000		0.000		0.000		0.000		0.000		0.001	*
	(0)		(0)		(0)		(0)		(0)		(0)	
Economic cycle	0.033		0.000		−0.023		−0.048	*	−0.034		0.067	*
	(0.018)		(0.03)		(0.035)		(0.021)		(0.021)		(0.023)	
Survey year												
2005	−0.007	**	−0.006	**	−0.006	**	−0.006	**	−0.007	**	−0.007	**
	(0.001)		(0.001)		(0.001)		(0.001)		(0.001)		(0.001)	
2006	0.006		0.016		0.023		0.031	**	0.024	**	−0.005	
	(0.006)		(0.008)		(0.011)		(0.007)		(0.007)		(0.007)	
2007	0.029	**	0.042	**	0.051	**	0.062	**	0.052	**	0.013	
	(0.008)		(0.009)		(0.015)		(0.01)		(0.01)		(0.008)	
2008	0.036	**	0.050	**	0.062	**	0.073	**	0.060	**	0.019	
	(0.008)		(0.011)		(0.017)		(0.011)		(0.011)		(0.01)	
2009	0.039	**	0.049	**	0.059	**	0.067	**	0.054	**	0.024	**
	(0.006)		(0.008)		(0.013)		(0.009)		(0.009)		(0.007)	
2010	0.019	**	0.023	**	0.030	*	0.035	**	0.023	*	0.006	
	(0.005)		(0.005)		(0.011)		(0.007)		(0.008)		(0.006)	
2011	0.004		0.007		0.016		0.020	*	0.006		−0.009	
	(0.006)		(0.006)		(0.012)		(0.008)		(0.009)		(0.007)	
2012	−0.020	**	−0.018	*	−0.009		−0.005		−0.021	*	−0.035	**
	(0.006)		(0.006)		(0.013)		(0.009)		(0.009)		(0.007)	
2013	−0.050	**	−0.054	**	−0.048	**	−0.048	**	−0.063	**	−0.061	**
	(0.007)		(0.008)		(0.012)		(0.008)		(0.009)		(0.008)	
2015	−0.059	**	−0.061	**	−0.053	**	−0.050	**	−0.068	**	−0.075	**
	(0.008)		(0.008)		(0.014)		(0.009)		(0.01)		(0.009)	
2015	−0.066	**	−0.066	**	−0.055	**	−0.050	**	−0.071	**	−0.084	**
	(0.009)		(0.009)		(0.016)		(0.011)		(0.012)		(0.01)	
2016	−0.043	**	−0.038	**	−0.022		−0.015		−0.040	*	−0.067	**
	(0.01)		(0.01)		(0.02)		(0.014)		(0.015)		(0.012)	
2017	−0.031	*	−0.021		0.000		0.012		−0.017		−0.062	**
	(0.012)		(0.013)		(0.025)		(0.017)		(0.018)		(0.014)	

Table A2 Analysis of effects on wages, full models (part 2)

	working independently		working to plan		alertness		explaining		collaboration		information gathering	
	b (*SE*)	*p*	*b* (*SE*)	*p*	*b* (*SE*)	*p*	*b* (*SE*)	*p*	*b* (*SE*)	*p*	*b* (*SE*)	*p*
Gender (female)	−0.052	**	−0.051	**	−0.052	**	−0.052	**	−0.051	**	−0.052	**
	(0.003)		(0.003)		(0.003)		(0.003)		(0.003)		(0.003)	
Age	0.016	**	0.016	**	0.016	**	0.016	**	0.016	**	0.016	**
	(0.001)		(0.001)		(0.001)		(0.001)		(0.001)		(0.001)	
Broad field of study (ref: economics)												
agriculture and food	−0.068	**	−0.068	**	−0.065	**	−0.065	**	−0.067	**	−0.067	**
	(0.007)		(0.007)		(0.007)		(0.007)		(0.007)		(0.008)	
teaching and education	−0.079	**	−0.079	**	−0.079	**	−0.080	**	−0.080	**	−0.077	**
	(0.011)		(0.011)		(0.011)		(0.011)		(0.011)		(0.011)	
technical	−0.057	**	−0.058	**	−0.053	**	−0.054	**	−0.057	**	−0.057	**
	(0.005)		(0.005)		(0.005)		(0.005)		(0.005)		(0.005)	
health studies	−0.065	**	−0.065	**	−0.062	**	−0.063	**	−0.065	**	−0.065	**
	(0.005)		(0.005)		(0.005)		(0.005)		(0.005)		(0.005)	
social studies	0.036	**	0.036	**	0.039	**	0.037	**	0.036	**	0.037	**
	(0.005)		(0.005)		(0.005)		(0.005)		(0.005)		(0.004)	
Region (ref: west)												
north	−0.027	**	−0.027	**	−0.027	**	−0.028	**	−0.028	**	−0.027	**
	(0.006)		(0.006)		(0.006)		(0.006)		(0.006)		(0.006)	
east	−0.005		−0.005		−0.005		−0.005		−0.005		−0.005	
	(0.003)		(0.003)		(0.003)		(0.003)		(0.003)		(0.003)	
south	0.022	**	0.022	**	0.023	**	0.022	**	0.022	**	0.022	**
	(0.002)		(0.002)		(0.002)		(0.002)		(0.002)		(0.002)	
Migration background												
western (excl. native	−0.001		−0.002		−0.002		−0.002		−0.001		−0.002	
	(0.004)		(0.005)		(0.005)		(0.005)		(0.005)		(0.005)	
non-western	0.014	**	0.014	**	0.013	**	0.013	**	0.014	**	0.014	**
	(0.003)		(0.003)		(0.003)		(0.003)		(0.003)		(0.003)	
Constant	2.637	**	2.654	**	2.603	**	2.594	**	2.657	**	2.641	**
	(0.006)		(0.007)		(0.015)		(0.009)		(0.011)		(0.006)	
R^2	0.106		0.105		0.108		0.108		0.105		0.186	

Table A2 Analysis of effects on wages, full models (part 3)

	creativity		learning ability		logical reasoning		occupation-specific knowledge		interdisciplinary knowledge		digital literacy	
	b (*SE*)	*p*	*b* (*SE*)	*p*	*b* (*SE*)	*p*	*b* (*SE*)	*p*	*b* (*SE*)	*p*	*b* (*SE*)	*p*
Required skill	0.004	*	0.004	**	0.007	**	0.014	**	0.006	**	0.002	
	(0.002)		(0.001)		(0.002)		(0.001)		(0.002)		(0.001)	
Linear trend	0.000		0.001	**	0.001	**	0.000		0.000		0.001	*
	(0)		(0)		(0)		(0)		(0)		(0)	
Economic cycle	−0.015		0.008		0.019		−0.026		0.002		0.046	
	(0.029)		(0.015)		(0.029)		(0.018)		(0.023)		(0.034)	
Survey year												
2005	−0.007	**	−0.009	**	−0.008	**	−0.005	**	−0.003	**	−0.006	**
	(0.001)		(0.001)		(0.001)		(0.001)		(0.001)		(0.001)	
2006	0.018	*	0.009	*	0.006		0.022	**	0.019	**	0.002	
	(0.008)		(0.004)		(0.008)		(0.004)		(0.005)		(0.008)	
2007	0.044	**	0.033	**	0.028	*	0.050	**	0.046	**	0.024	*
	(0.01)		(0.005)		(0.01)		(0.005)		(0.006)		(0.01)	
2008	0.053	**	0.039	**	0.034	**	0.059	**	0.057	**	0.031	**
	(0.011)		(0.005)		(0.011)		(0.006)		(0.006)		(0.01)	
2009	0.049	**	0.037	**	0.033	**	0.053	**	0.056	**	0.033	**
	(0.008)		(0.003)		(0.008)		(0.004)		(0.004)		(0.006)	
2010	0.020	**	0.010	**	0.008		0.023	**	0.032	**	0.012	**
	(0.006)		(0.002)		(0.006)		(0.002)		(0.003)		(0.004)	
2011	0.003		−0.008	**	−0.010		0.006	*	0.018	**	−0.004	
	(0.006)		(0.002)		(0.006)		(0.003)		(0.004)		(0.005)	
2012	−0.023	**	−0.034	**	−0.037	**	−0.020	**	−0.007		−0.029	**
	(0.006)		(0.002)		(0.007)		(0.004)		(0.005)		(0.006)	
2013	−0.061	**	−0.071	**	−0.074	**	−0.060	**	−0.040	**	−0.058	**
	(0.007)		(0.005)		(0.01)		(0.006)		(0.007)		(0.01)	
2015	−0.068	**	−0.080	**	−0.084	**	−0.066	**	−0.046	**	−0.069	**
	(0.008)		(0.004)		(0.01)		(0.006)		(0.007)		(0.009)	
2015	−0.073	**	−0.088	**	−0.092	**	−0.069	**	−0.049	**	−0.077	**
	(0.008)		(0.004)		(0.01)		(0.006)		(0.007)		(0.009)	
2016	−0.044	**	−0.064	**	−0.070	**	−0.038	**	−0.020	**	−0.056	**
	(0.01)		(0.003)		(0.012)		(0.005)		(0.006)		(0.008)	
2017	−0.025		−0.049	**	−0.057	**	−0.017	**	−0.003		−0.048	**
	(0.013)		(0.004)		(0.014)		(0.006)		(0.007)		(0.01)	

Table A2 Analysis of effects on wages, full models (part 4)

	creativity		learning ability		logical reasoning		occupation-specific knowledge		interdisciplinary knowledge		digital literacy	
	b *(SE)*	*p*	*b* *(SE)*	*p*	*b* *(SE)*	*p*	*b* *(SE)*	*p*	*b* *(SE)*	*p*	*b* *(SE)*	*p*
Gender (female)	−0.051	**	−0.052	**	−0.051	**	−0.051	**	−0.051	**	−0.051	**
	(0.003)		(0.003)		(0.003)		(0.003)		(0.003)		(0.003)	
Age	0.016	**	0.016	**	0.016	**	0.016	**	0.016	**	0.016	**
	(0.001)		(0.001)		(0.001)		(0.001)		(0.001)		(0.001)	
Broad field of study (ref: economics)												
agriculture and food	−0.067	**	−0.068	**	−0.068	**	−0.068	**	−0.068	**	−0.069	**
	(0.007)		(0.007)		(0.007)		(0.007)		(0.007)		(0.007)	
teaching and education	−0.080	**	−0.080	**	−0.078	**	−0.082	**	−0.080	**	−0.081	**
	(0.011)		(0.011)		(0.011)		(0.011)		(0.011)		(0.011)	
technical	−0.058	**	−0.058	**	−0.058	**	−0.056	**	−0.057	**	−0.059	**
	(0.005)		(0.005)		(0.005)		(0.005)		(0.005)		(0.005)	
health studies	−0.065	**	−0.065	**	−0.065	**	−0.063	**	−0.065	**	−0.067	**
	(0.005)		(0.005)		(0.005)		(0.005)		(0.005)		(0.005)	
social studies	0.036	**	0.035	**	0.035	**	0.034	**	0.036	**	0.035	**
	(0.005)		(0.005)		(0.005)		(0.004)		(0.004)		(0.005)	
Region (ref: west)												
north	−0.028	**	−0.028	**	−0.027	**	−0.027	**	−0.027	**	−0.027	**
	(0.006)		(0.006)		(0.006)		(0.006)		(0.006)		(0.006)	
east	−0.005		−0.005		−0.005		−0.005		−0.004		−0.005	
	(0.003)		(0.003)		(0.003)		(0.003)		(0.003)		(0.003)	
south	0.022	**	0.022	**	0.023	**	0.022	**	0.023	**	0.022	**
	(0.002)		(0.002)		(0.002)		(0.002)		(0.002)		(0.002)	
Migration background (ref: native Dutch)												
western (excl. native	−0.002		−0.002		−0.001		−0.001		−0.001		−0.002	
	(0.005)		(0.005)		(0.005)		(0.005)		(0.005)		(0.005)	
non-western	0.014	**	0.013	**	0.014	**	0.014	**	0.014	**	0.013	**
	(0.003)		(0.003)		(0.003)		(0.003)		(0.003)		(0.003)	
Constant	2.650	**	2.652	**	2.638	**	2.610	**	2.641	**	2.659	**
	(0.007)		(0.004)		(0.007)		(0.005)		(0.005)		(0.006)	
R^2	0.105		0.107		0.010		0.108		0.106		0.106	

* p < 0.05, ** p < 0.01.
